# Assessing the Effects of Indigenous Migration on Zootherapeutic Practices in the Semiarid Region of Brazil

**DOI:** 10.1371/journal.pone.0146657

**Published:** 2016-01-08

**Authors:** Carlos Alberto Batista Santos, Ulysses Paulino de Albuquerque, Wedson Medeiros Silva Souto, Rômulo Romeu Nóbrega Alves

**Affiliations:** 1 Programa de Pós Graduação em Etnobiologia e Conservação da Natureza, Departamento de Ciências Biológicas, Universidade Federal Rural de Pernambuco, Rua Dom Manoel de Medeiros, s/n, Dois Irmãos, 52171–900, Recife, PE, Brasil; 2 Departamento de Tecnologia e Ciências Sociais, Universidade do Estado da Bahia, Avenida Edgard Chastinet, s/n, São Geraldo, 48905–680, Juazeiro, BA, Brasil; 3 Universidade Federal do Piauí, BR 343, km 3,5, Bairro Meladão, 64800–000, Floriano, PI, Brasil; 4 Programa de Pós-Graduação em Ciências Biológicas (Zoologia), Departamento de Sistemática e Ecologia, Centro de Ciências Exatas e da Natureza, Universidade Federal da Paraíba (UFPB), Campus I, João Pessoa, PB, 58051–900, Brasil; 5 Departamento de Biologia, Universidade Estadual da Paraíba, Av. das Baraúnas, 351/Campus Universitário, Bodocongó, 58109–753, Campina Grande, PB, Brasil; University of Campinas, BRAZIL

## Abstract

Human migration implies adaptations to new environments, such as ways to benefit from the available biodiversity. This study focused on the use of animal-derived remedies, and we investigated the effects of migration on the traditional medical system of the indigenous Truká people. This ethnic group lives in Northeast Brazil and is currently distributed in four distinct villages. In these villages, the zootherapeutic knowledge of 54 indigenous people was determined through semi-structured questionnaires given from September 2013 to January 2014. The interviewees indicated 137 zootherapeutic uses involving 21 animal species. The variety of species and their uses have a higher similarity between villages that are closer to each other, which can be a reflection of geographic and environmental factors. However, even close villages showed a low similarity in the zootherapeutic uses recorded, which reflects a strong idiosyncrasy regarding the knowledge of each village. Hence, each village may be influenced by the physical environment and contact with other cultures, which may maintain or reduce the contact of younger villages with the original village.

## Introduction

One of the reasons that causes humans to leave their place of origin is the search for available natural resources for their subsistence [1,2], which has resulted in the migration of human communities throughout history, not only to other rural areas but also to cities [3,4]. In the last decade, human migration throughout the world has surpassed 230 million people [5], driving numerous human groups to different environments, even within their own country of origin.

The migratory process obviously implies adaptations to newly occupied environments, such as ways to benefit from available biodiversity. Contact with the new environment allows an incorporation of new biological resources, causing an alteration in the diversity of animals and plants of useful value known by these migrants [6]. Research on the use of medicinal plants by migrating populations, for example, reveals that the diversity of plants is altered due to their use. In certain cases, the adjustment results in the addition of new medicinal species and, in others, obtaining these species from their place of origin [1,2,6–12]. Information about migratory movements in certain countries is scarce, even when considering traditional populations that have undergone small-scale migratory processes. In this scenario, the present research investigated the effects of regional migration on the richness and diversity of the animals used traditional folk medicine in the northeast region of Brazil.

In Brazil, historical documents and recent studies reveal that several animal species have been used for medicinal purposes by indigenous societies and by numerous Europeans and Africans who arrived during colonial times [13–16]). The interaction between these diverse cultural elements formed the basis of the Brazilian culture, which reflects the country’s traditional medicine popularly used by numerous communities as the main source for treating health problems [14,17,18].

Particularly in the semiarid region of northeastern Brazil, animal by-products are used by traditional and indigenous communities for treating diseases and disorders in several locations [19], such as traditional communities and local indigenous tribes that have been using animals for these purposes throughout their history [20–22]. The importance of zootherapeutic products in traditional medicine in the northeast region has been described in several recent studies of rural and urban areas [15,19,23–27]. However, little research has focused on the traditional medicine practiced by the indigenous communities of the region [28,29]. Several of these communities experienced significant alterations in their territories during the colonization process of the northeast region of Brazil. As a consequence, the current distribution of indigenous communities was influenced by slavery and genocide, as well as by the invasion of their territories, which resulted in the migration of various ethnic groups displaced from their place of origin [30].

By virtue of this scenario, the present study investigated the influence of the migratory process on the use of animals used traditional medicine by indigenous people inhabiting of the Northeast region of Brazil. The research involved the Truká people, an indigenous group that inhabits the semiarid northeast region of Brazil that migrated dissimilar distances to different areas within the region. It is hypothesized that the use of medicinal animals by different migrant groups was affected by the influence of the new environments and resulted in the alteration of the region’s zootherapeutic arsenal. It was expected that there would be changes in the patterns of the zootherapeutic species and their respective therapeutic uses by the migrant populations of the same macro-region (semiarid region of northeastern Brazil), with a higher similarity between close villages with similar environmental conditions.

## Methods

### Research area and the Truká indigenous people

The research was conducted in the four Truká villages in Northeast Brazil. Two of them are located in the state of Pernambuco: the Central Village in the municipality of Cabrobó (8° 31’ 07.11” S x 39° 22’ 20.87” W) and the other village in the municipality of Orocó (8° 36’ 24.4” S x 39° 34’ 54.9” W). The other villages studied are located in the state of Bahia, one in the municipality of Paulo Afonso (9° 25’ 10.58” S x 38° 16’ 31.05” W) and the other in the municipality of Sobradinho (9° 29’ 47.7” S x 40° 51’ 7.9” W). Assunção Island in Pernambuco’s municipality of Cabrobó is designated by the Truká people as the Central Village, because it originated from the migration of Truká villages from other cities of Pernambuco’s and Bahia’s *sertão* [31].

**Fig 1:**
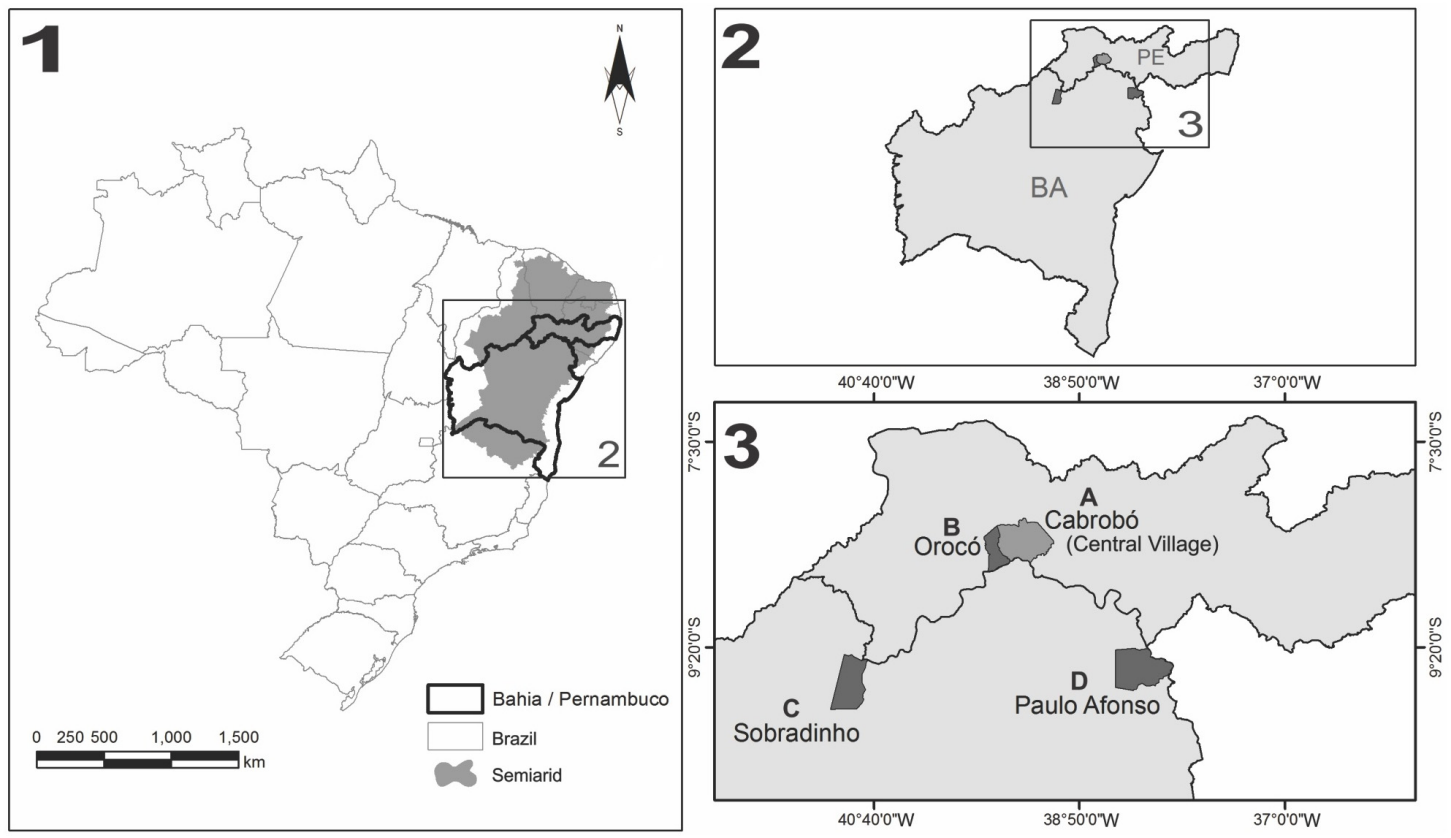
Map showing the location of the study areas indicating the Truká villages in the Brazilian semiarid region.

All of the locations studied are situated in the semiarid northeastern region **(**Fig 1) in the lower middle São Francisco. The Orocó, Paulo Afonso and Sobradinho villages are distant from the Central Village (Cabrobó), 39.85, 211.8 and 239.18 km, respectively.

The study areas feature typical Caatinga vegetation, where agriculture is the main economic activity, besides livestock and craftwork as secondary means of income.

The municipalities of Orocó and Cabrobó are located in the São Francisco mesoregion and the Petrolina microregion of the state of Pernambuco, included in the geoenvironmental unit of the “Sertaneja Depression,” which contains a typical northeastern semiarid landscape, characterized by a monotonous pediplanation surface, with a predominantly slightly undulating terrain, divided by narrow valleys with dissected strands. The vegetation is basically composed of xerophytic Caatinga with stretches of deciduous forest. The climate is of the semiarid tropical type, with rain in the summer. The rainy season starts in November and ends in April, and the average precipitation is 431.8 mm [32,33].

The municipality of Sobradinho, state of Bahia, is part of the Drought Polygon, with an arid climate and average annual temperature of 27°C, and the annual average rainfall is between 400 and 500 mm, with a high probability of prolonged drought. The predominant vegetation is open or dense Caatinga and park land, with no palm trees. Drained by the São Francisco River, the Sobradinho Lake is the largest artificial lake in Brazil [34].

The municipality of Paulo Afonso, also included in the Drought Polygon in Bahias’s backcountry, has a semiarid and arid megathermal climate, with an average annual temperature of 29.1°C and average annual rainfall of 907 mm, with the rainy season occurring between May and July. It contains rounded hills and fluvial plains drained by the São Francisco River and tributaries. The native vegetation is characterized by open arboreal Caatinga (seasonal dry forest), with or without palm trees and contact between Cerrado-Caatinga seasonal forests [35].

### Data collection

The data were collected between September 2013 and January 2014. Authors stayed four days a month in each village, i.e., a total of 20 days/village. The information was gathered from 54 interviewees (37 males and 17 females; median age 55. 7—range 18–71)); 16 were interviewed in the Central Village (Cabrobó), 12 in Orocó, 12 in Sobradinho and 14 in Paulo Afonso.

The sampling method was based on the non-probabilistic intention type [36], and the snowball sampling technique [37] was used to find possible interviewees. The sampling included the following: caciques, Truká village chiefs who organize, speak for, guide and represent the people or the village, above all others; pajés, medical and spiritual leaders; benzedeiras, women who pray to heal the diseases of the body and soul; shrine chiefs, preparers of the conditions for the practices of traditional medicine and religion; and jurumeiros, specialists in the preparation of the jurema. *Mimosa tenuiflora* (Willd.) Poir is a native tree that has a symbolic value and is considered an enchanted being that represents the forces of nature [38]. It is used in the preparation of jurema wine, and its ingestion allows a connection with the ancestors who reveal the secrets of the indigenous science used to heal the body and the soul [38]. All interviewees spoke Portuguese, so all interviews were conducted in this language.

The information about the use of animals for medicinal purposes was collected through semi-structured questionnaires with the use of informal interviews and conversations to gather complementary data [36,39]. The questionnaires comprised questions about the animal species used for medicinal purposes, health problems treated, and preparations and uses of the medicines.

### Identification of the animal species

The vernacular names of the species were recorded as cited by the people interviewed. In many cases, the identification of the animals was established by directly examining the whole animal or their usable parts during the interviews. In general, the animals were identified in the following ways: 1) analysis of specimens donated by the interviewees; 2) analysis of photographs of animals (or of their parts) done during the interviews; and 3) through vernacular names, with the help of taxonomists familiar with the fauna of the study area. In the few cases where identification was not possible by one of the aforementioned methods, animal parts or the entire animal (small-sized ones such as termites or other insects) of each species was collected for later identification. Samples were stored in the Training and Indigenous Research Center of Bahia State University.

### Ethics statement

Regarding the ethical aspects of this research, the purposes of the present study were explained before each interview. Likewise, permission was obtained to record the information by signing an informed consent form (ICF) and giving authorization for using images. The ethical approval for the study was obtained from the Ethic Committee of Bahia State University (Protocol No. 723.750). Authorization to access the traditional knowledge associated with genetic heritage was granted by the National Historic and Artistic Heritage Institute (No. 013/2013. legal process No. 01450.010527/2013-30). The clearance to enter indigenous territories was granted by the National Indigenous Foundation, which is supported by the Lower São Francisco Regional Coordination.

### Data analysis

Incidence matrices (of use/non-use) of zootherapeutic species were prepared for each village using the software Libre Office Calc v. 4.2. The binary matrix of the species used in each village was used to create a single binary matrix with all the richness of zooterapeutic species exploited (or not) in all villages. According to this matrix, the different Truká villages were evaluated for their simililarity in terms of patterns of use of zootherapeutics, which allowed us to determine if the villages formed by the migrants that are closer to the Central Village (Cabrobó) are more similar to it, in relation to the species and their respective medicinal uses. MRPP (multiple response permutation procedure) was performed to determine if there were any differences between the areas with regard to repertoire of species and their uses. The test was performed with the MRPP function in the Vegan package of R software, using 1000 permutations and the Jaccard distance (Jaccard similarity) [40]. Basically, test compares the mean dissimilarity witin each group with the mean similarity between all combinations. If there were groups with less internal than external dissimilarity, then there would be differences between the groups. Using this matrix, Jaccard was used to evaluate how similar, in terms of patterns of use of zootherapeutics, the different Truká villages were, allowing us to determine if the villages formed by the migrants that were closer to Central Village (Cabrobó) were more similar to it, in relation to the species used and their respectives uses.

The non-parametric Kruskal-Wallis test [41, 42] was used to compare species richness between the locations studied and the number of zootherapeutic uses indicated by each interviewee; this allowed testing the hypothesis that migrant villages do not completely abandon their traditional practices and do not differ significantly in number of species and zootherapeutic uses cited by each sample unit (interviewee). When necessary, the Mann-Whitney *post-hoc* test was used [43]. These tests were carried out with the help of Statistica v.10 software.

## Results and Discussion

**Fig 2:**
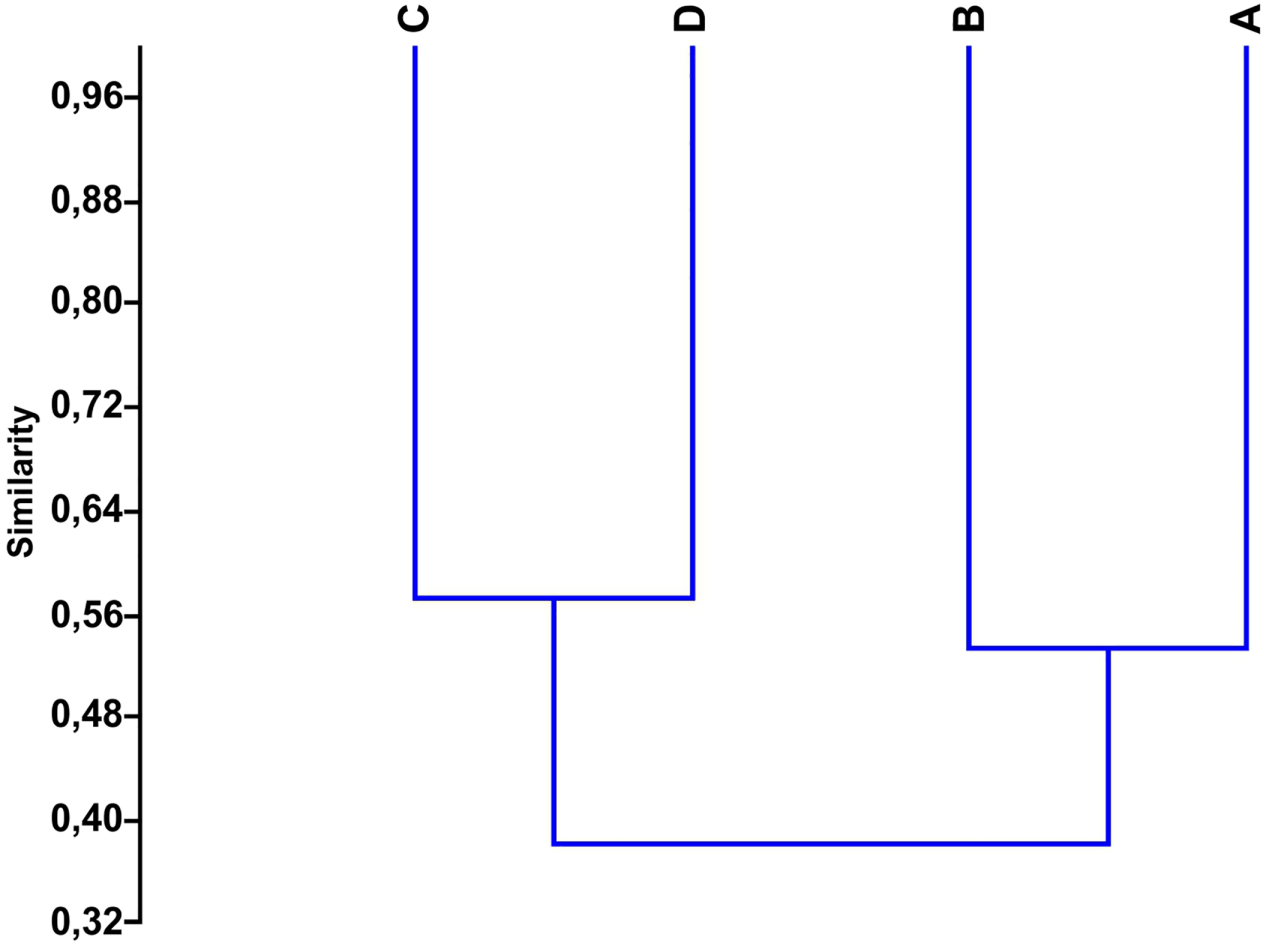
Grouping analysis using Jaccard’s similarity index of the variety of species used for medicinal purposes in the four Truká villages. The Central Village is closer to the Orocó village (J = 0.53), and the Sobradinho village is closer to the Paulo Afonso village (J = 0.57). A: Central Village (Cabrobó), B: Orocó, C: Sobradinho, D: Paulo Afonso.

In the four villages, a total of 21 animal species were cited (Table 1). Considering the variety of species between the villages, an analysis of similarities revealed two distinct groups as follows: one composed of the Central Village (Cabrobó) and the Orocó village (Jaccard´s similarity index, J = 0.53) and another composed of the Sobradinho and Paulo Afonso villages (J = 0.57) (Fig 2). Indeed, the latter two villages were more similar to each other, but the Paulo Afonso village was the most dissimilar with respect to all villages and surpassed the total average distance determined (Table 2). These results confirmed our expectation that the villages located in proximity tended to have more similarities with regard to the variety of animal species used in traditional medicine. The groups established in this study may be a reflection of geographic and environmental factors because the first two villages are geographically close to each other (separated by only 39.85 km). In addition, they are located in the lower medium São Francisco region, where xerophytic Caatinga vegetation predominates, with stretches of deciduous forests [32,34]. Wildlife surveys conducted by the Caatinga Conservation and Fauna Management Center [44] showed the common occurrence of animal species between the two Pernambuco municipalities, where these villages are located. Hence, the use of the same species by the migrants of the Orocó village in traditional medicine was expected because the available fauna is similar to that found in the Central Village.

**Table 1:** Animal species used for medicinal purposes by the Truká people in the semiarid region of northeastern Brazil.

Family/species/local name (common name)	Number of citations	Part used and administration form	Citations (villages)				Disease
			CA	OR	SO	PA	
**INSECTS**							
*Gryllus assimilis* Fabricius, 1775—cricket, “grilo”	1	Hind legs (1)			1		Kidney inflammation
Apidae							
*Melipona scutellaris* (Latreille, 1811)—Stingless bee, “abelha urucú”	1	Wax (1)				1	Sore throat and flu
**FISHES**							
Erythrinidae							
*Hoplias malabaricus* (Bloch, 1794)—Trahira, “traíra”	6	Fat (2)	2	3			Earache, toothache and fatigue
Pimelodidae							
*Pseudoplatystoma corruscans* (Spix & Agassiz,1829)–spotted sorubim, “surubim”	2	Spine (3)	1	1			Remove wrath and evil eye
**REPTILES**							
Boidae							
*Boa constrictor* (Linnaeus, 1758)–Boa snake, “jibóia”	9	Fat (2)	2	3	3	1	Cracked feet, remove slivers, remove thorns, inflammation, rheumatism, pain, maimedness, joint pain and back pain.
*Epicrates assisi* (Machado, 1945)—Brazilian rainbow boa, “salamanta”	4	Fat (2)	3	2			Remove slivers, remove thorns and leg pain
Viperidae							
*Crotalus durissus* (Linnaeus, 1758)—Neotropical rattlesnake, “cascavel”	11	Fat (2)	2	4	2	1	Back pain, toothache, nasal congestion, inflammation, rheumatism, remove slivers, remove thorns, pain, maimedness, earache, sore throat and remove splinters
Aligatoridae							
*Caiman latirostris* (Daudin, 1802)–Broad-snouted caiman, “jacaré-do-papo-amarelo”	35	Hide (3) (4) (8) (11), Fat (2), Skin (3) (4), Teeth (5), Meat (6), Nails (2)	12	10	7	2	Chase away evil, headaches, pain, stroke, evil eye, toothache, fever, epilepsy, rheumatism, break spells, bone pain, free the body of spirits, inflammation, brain deformity, fatigue, seal the body from spirits, sore throat, remove evil spirits, muscle pain, vomiting, remove thorns, stomach ache, CVA, allergies, nasal polyps, constipation, nose bleeds e tooth eruption.
Iguanidae							
*Iguana iguana* (Linnaeus, 1758)—Common iguana, “camaleão”	12	Fat (2)	3	2	2	3	Remove slivers, remove thorns, tumor, rheumatism, leg pain, joint pain, tuberculosis and evil eye
Teiidae							
*Tupinambis merianae* (Duméril andBibron, 1839)—Tegu lizard, “teíu”	17	Fat (2)	6	3	3	7	Inflammation, earache, tumor, foot wounds, pain, headache, Cracked feet, flu, sore throat and throat inflammation
Chelidae							
*Phrynops geoffroanus* (Scweigger, 1812)–Geoffroy’s side-necked turtle, “cágado”	4	Shell (3) and Fat (2)	2	3			Rheumatism, leg pain and evil eye
**AVES**							
Phasianidae							
*Gallus domesticus* (Linnaeus, 1758)–Domestic chicken, “galinha”	18	Fat (2), Oil (2) (7) and feces (8)	3	4	8	6	Burns, inflammation, chase away evil, weakness, pain, flu, sore throat, earache, nasal congestion, wounds, throat inflammation, headache, grow hair, baldness, nasal decongestion
Anatidae							
*Cairina moschata* (Linnaeus, 1758)–Muschovy duck, pato	1	Egg (7)				1	Weakness
**MAMMALS**							
Felidae							
*Puma yagouaroundi* (E. Geoffroy Saint-Hilare, 1803)–Jaguarundi, “gato vermelho”	**1**	Hide (3)			1		Asthma
Cervidae							
*Mazama gouazoupira* (Fischer, 1914)–Gray brocket, “veado-catingueiro”	1	hooves (3)			1		Sore throat
Hidrochaeridae							
*Hydrochoerus hidrochaeris* (Linnaeus, 1766)–Capybara, “capivara	14	Fat (2), Bone (3) and Oil (7)	11	2			Dislocations, toothache, rheumatism, bone pain, joint pain, chase away evil, free the body of spirits, burns, blows, strokes and inflammations
Dasypodidae							
*Euphractus sexcinctus* (Linnaeus, 1758)—Six-Banded Armadillo, “tatu peba”	2	Meat (6) and Tail (9)	1				Asthma and earache
Bovidae							
*Ovis aries* (Linnaeus, 1758)—Sheep, “carneiro”	8	Fat (2) and Tallow (2)		4	5	1	Nerves, leg pain, joint pain, remove slivers, cracked feet, rheumatism, muscle pain, weakness, back pain, remove splinters, blows, knee pain and swelling
*Bos taurus* (Linnaeus, 1758)–Bull, Cow, “boi”, “vaca”	12	Horns (10), Butter (2) (6) and Calf’s-foot jelly (6)	2	2	3	3	Tumor, sore throat, congested nose, cough, weakness, evil eye, repel snakes, cracked feet and burns
Suidae							
*Sus scrofa* (Linnaeus, 1758)—Pig, “porco”	2	Feces (8)	1				Leg pain and evil eye
Equidae							
*Equus asinus* Linnaeus, 1758—Female donkey, “jumenta”	5	Milk (7)	2			2	Cough

Legend: CA = Cabrobó, OR = Orocó, SO = Sobradinho, PA = Paulo Afonso. In parts used: (1) Prepare tea with the part of the animal and ingest; (2) Rub on affected area; (3) Stomp, roast, prepare tea and ingest; (4) Use in a smoker; (5) tie with a red ribbon, hang on the neck, arm or carry in a purse or pocket (6) Cook and ingest; (7) Ingest pure, without cooking; (8) Roast and rub on affected area; (9) Place inside ear canal; (10) Burn in front of the house and store in the house entrance; (11) Tie to the ceiling or entrance of the house.

**Table 2:** Results of the multiple response permutation procedure about the variety of species and their medicinal uses in the four Truká villages in the northeast of Brazil.

Richness of Species				
Significance of delta (p): 0.000999				
Observed delta: 0.7688				
Expected delta: 0.8133				
Chance corrected within-group agreement A: 0.05475				
Villages	Central Village	Orocó	Sobradinho	Paulo Afonso
Delta	0.7087	0.7783	0.7513	0.8443
Weights for groups	16	12	12	14
Uses of the Species				
Significance of delta (p): 0.000999				
Observed delta: 0.9547				
Expected delta: 0.9714				
Chance corrected within-group agreement A: 0.01714				
Villages	Central Village	Orocó	Sobradinho	Paulo Afonso
Delta	0.9566	0.9825	0.9296	0.9503
Weights for groups	16	12	12	14

The fact that the Cabrobó (Central Village) and Orocó villages are located on islands of the São Francisco River should be highlighted because the access to aquatic animals is higher compared with villages of the second group (Sobradinho and Paulo Afonso villages). In this scenario, it was not surprising that aquatic species such as the trahira (*Hoplias malabaricus*) and the spotted sorubim catfish (*Pseudoplatystoma corruscans*) and semiaquatic species such as the toadhead turtle (*Phrynops geoffroanus*) and the capybara (*Hidrochaerus hidrochaeris*) were cited only in the Cabrobó and Orocó villages because of the easy access to these animals in those areas. These results confirm previous ethnozoological research findings [15,45,46] that demonstrated that the local fauna composition influences the selection of animal species used for medicinal purposes.

The second group was composed of the villages in the Bahia municipalities of Paulo Afonso and Sobradinho (J = 0.57; Fig 2), located in the Drought Polygon with Caatinga vegetation and similar physiognomy. With regard to the fauna, Sobradinho and Paulo Afonso had species in common with other areas of the neighboring northeastern Caatinga [47–52]. These two municipalities of Bahia had large areas of Caatinga flooded due to the construction of hydroelectric dams, which resulted in the reduction of habitats and, as a consequence, a reduction of the terrestrial fauna. In the last 60 years, large dams were constructed along the São Francisco River to produce hydroelectric energy, such as the Sobradinho Lake Hydroelectric Dam, the Moxotó Power Station and the Paulo Afonso Power Stations (I, II, III and IV) in the municipality of Paulo Afonso [53]. The construction of the last dams resulted in the violent eviction of the Indigenous people from their lands [54] and constituted the human intervention that most impacted the São Francisco River basin and its surroundings [55].

The caiman (*Caiman latirostris*) was the only aquatic species that had medicinal purposes recorded in all the villages. However, it was mostly cited in the villages near the São Francisco River, e.g., it was cited 12 times in the Central Village in Cabrobó, 10 in Orocó, and 7 in Sobradinho, but only twice in Paulo Afonso. These results suggest that this species has great importance in Truká zootherapy when considering the intensity and multiplicity of its uses, as well as its persistence in the memory and practices of local medicine, regardless of migration.

The medicinal fauna of a specific region, consisting mostly of local species, also included allochthonous animals [24] observed in the areas studied. The addition of allochthonous species in the medicinal arsenal of a particular area became possible due to the existence of commercial routes or human migration from one location to another. Moreover, people try to perpetuate their culture and the use of their medicinal products of certain animal and plant species, even though they occur in areas distant from their villages [15,17,25].

Likewise, the results of the present study suggest that the migrants of the Truká people adapted to the use of zootherapeutic resources available in the new locations to which they moved. For example, the use of crickets (*Gryllus assimilis*) for treating renal inflammation by the migrants of Sobradinho was a possible replacement for capybara (*Hydrochoerus hidrochaeris*) (Table 2), which are difficult to find in this area. In addition to the availability of animals in the environment, the addition of new zootherapeutic elements may be the result of contact with other human cultures. For instance, the territories of the Paulo Afonso and Sobradinho villages are surrounded by agricultural properties of non-indigenous populations, which may have allowed the exchange of zootherapeutic knowledge. Several studies have shown that contact between migrants and other indigenous and non-indigenous cultures may result in the addition and replacement of the natural resources used for medicinal or mystical, religious purposes [2,56,57].

**Fig 3:**
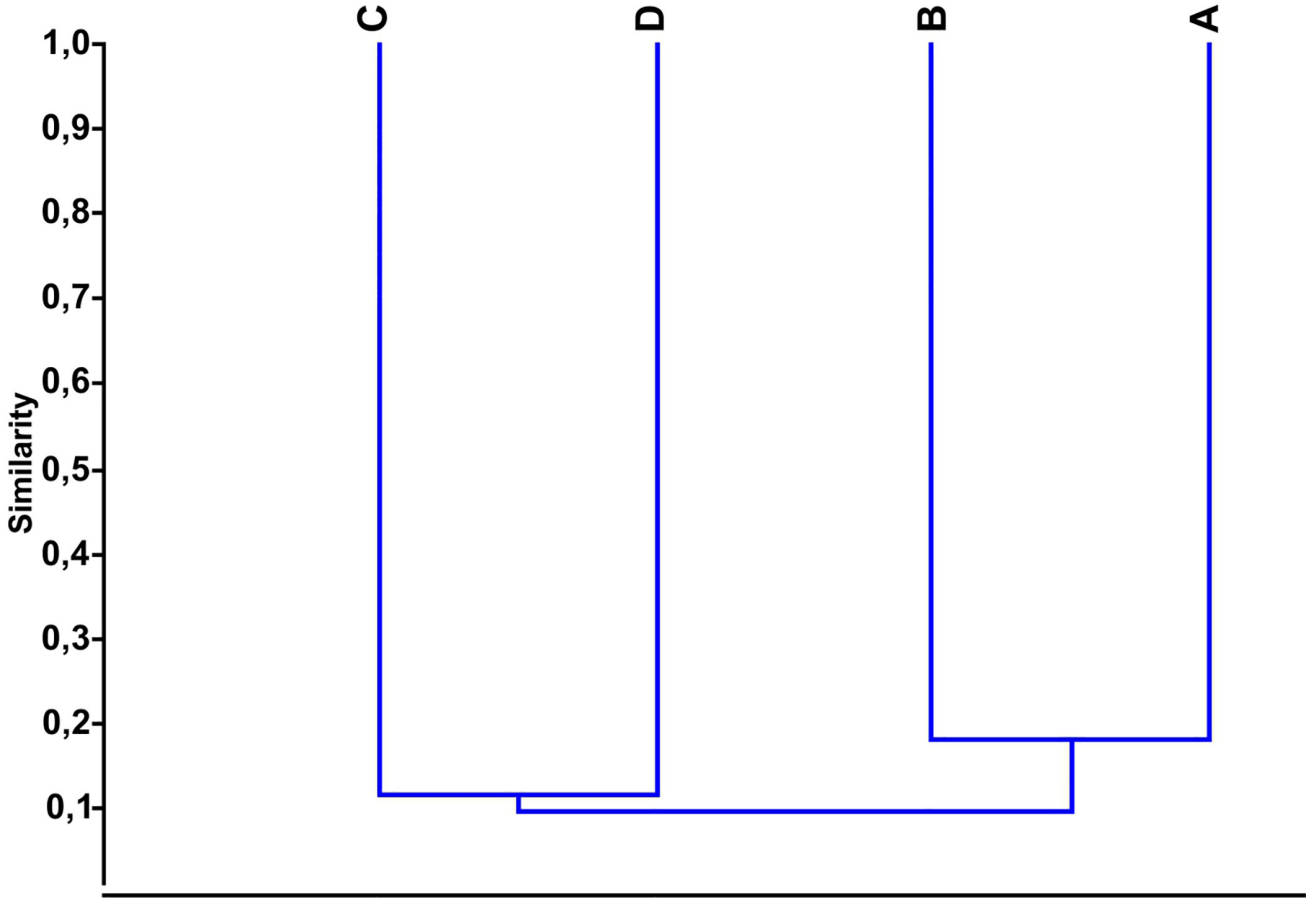
Cluster analysis using Jaccard’s similarity index and the composition of zootherapeutic uses in the four Truká villages. The Central Village is more related to the Orocó village (J = 0.18), and the Sobradinho village is closer to the Paulo Afonso village (J = 0.11). A: Central Village (Cabrobó), B: Orocó, C: Sobradinho, D: Paulo Afonso.

The similarity index of the 137 recorded zootherapeutic uses of the 21 animal species between the villages was low, and it varied from 0.09 to 0.18 (Fig 3). As with the cluster analysis according to the species used, the dendrogram generated from the similarities of zootherapeutic uses formed two groups, which demonstrated that the Central Village (Cabrobó) was more related to the Orocó village (J = 0.18), whereas the Sobradinho and Paulo Afonso villages were closer with regard to zootherapeutic uses (J = 0.11; Fig 3). Although there was similarity in the species used, the groups were heterogeneous with respect to the uses of these species (Table 2).

**Fig 4:**
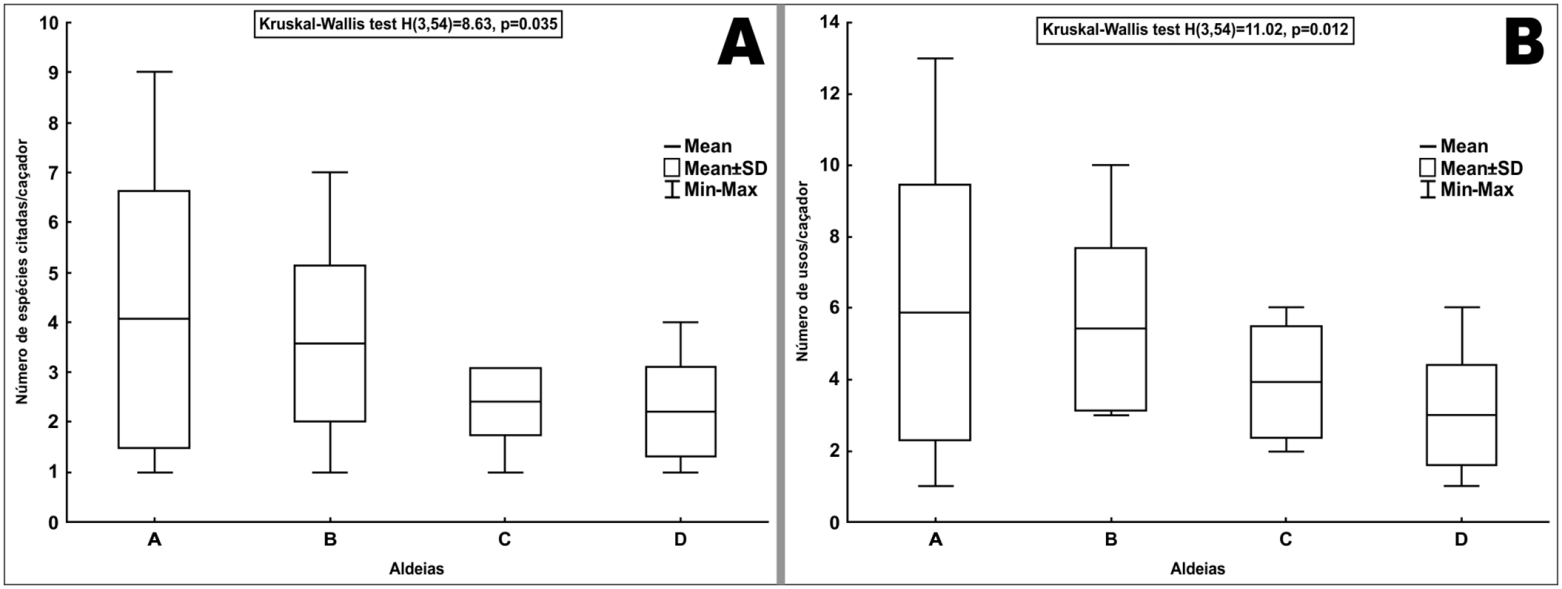
(A) Number of explored species by the Truká people in the four selected villages. (B) Number of zootherapeutic uses informed by each interviewee of the Truká villages. A—Central Village (Cabrobó), B—Orocó, C—Sobradinho, D—Paulo Afonso.

The number of zootherapeutic uses also showed substantial variation between the villages. The indigenous people from the Central Village (Cabrobó) cited a larger number of zootherapeutic uses (n = 59), followed by Orocó (n = 57), Sobradinho (n = 33) and Paulo Afonso (n = 25). A Kruskal-Wallis H test showed significant differences between the locations when comparing the number of reported uses by each interviewee (H_(3)_ = 11.02, p = 0.012; Fig 4). Mann-Whitney’s *post hoc* tests indicated that the Paulo Afonso indigenous people had a significantly smaller repertoire of zootherapeutic uses compared to the Central Village (Cabrobó; U = 52; p = 0.012, ΣR Cabrobó = 308; ΣR Paulo Afonso = 157) and the Orocó (U = 27; p = 0.002, ΣR Orocó = 219; ΣR Paulo Afonso = 132). The higher numbers of uses in the Central Village (Cabrobó) and Orocó village were the result of the higher number of medicinal species recorded in these two locations.

Little similarity in zootherapeutic uses was found between the Central Village (Cabrobó) and the Orocó village (J = 0.18). The similarity was low even though they are geographically close and have coexisted for a relatively long time, and their environmental characteristics are similar. The Sobradinho and Paulo Afonso villages are farther from the Central Village; they are younger and are more similar to each other (J = 0.11); however, their similarity index was low. This pattern indicates that knowledge about animals has a high idiosyncrasy, which indicates that the people who established the younger villages have a different knowledge than those from the Central Village. A similar situation was observed by Vandebroek [58], who compared the traditional medicinal plant knowledge between two groups of traditional witchdoctors from the Andes and the Bolivian Amazon; she concluded that knowledge is acquired individually in an idiosyncratic way through plant experimentation and may or may not be shared in a certain way with others. The results of the present study suggest that a similar situation occurred in the area surveyed with regard to the knowledge of medicinal animals because a low similarity was found between the villages studied. Other factors may influence the difference in medicinal knowledge between the villages, such as the fauna composition in each location [15,24, 59] and the effect of the contact between the villages and urban, non-indigenous communities (e.g., Paulo Afonso and Sobradinho villages). These situations allow the diversification of the local medical system [2,56, 57, 60] and the replacement of the animal resources used in traditional medicine by plant-based or allopathic medications.

## Conclusions

Zootherapeutic practices of the Truká people persisted as a therapeutic alternative in all of the villages studied. However, certain variations occurred in the repertoire of medicinal species and their respective uses between the Truká migrants of the villages compared. Instead, there were minimal differences between the Truká villages of the municipality of Paulo Afonso and Sobradinho compared with the Central Village (Cabrobó).

The contact between the migrant people who moved to places near urban areas, such as the Paulo Afonso and Sobradinho villages in Bahia, allowed the exchange of medicinal experience, which altered the zootherapeutic arsenal with the incorporation of new medicinal species that compensated for the loss of other previously used species. Moreover, this cultural contact resulted in the familiarization with and integration of allopathic medicine, which could have led to a gradual reduction in zootherapeutic use.

Another important factor was the diminishing contact between the Paulo Afonso and Sobradinho villages with their place of origin, which was enhanced by the distance between their indigenous territories. Closer communities established in environments with similar characteristics, such as the Central Village and the Orocó village, allowed the exchange of experiences and information between villages.

Each Truká village showed idiosyncratic knowledge about medicinal animals, which was certainly influenced by the physical environment, by contact with other cultures and by maintaining or decreasing contact with the Central Village of Cabrobó, the place of origin of all of these people. It is necessary to investigate other aspects of the cultural use of wild fauna by the Truká ethnic group to verify whether or not these factors interact and influence the uses of natural resources by migrant populations.
